# Relationships between academic self-efficacy, learning-related emotions, and metacognitive learning strategies with academic performance in medical students: a structural equation model

**DOI:** 10.1186/s12909-020-01995-9

**Published:** 2020-03-17

**Authors:** Ali Asghar Hayat, Karim Shateri, Mitra Amini, Nasrin Shokrpour

**Affiliations:** 1 Clinical Education Research Center, Shiraz University of Medical Sciences, Shiraz, Iran; 2 Department of primary education, Abdanan center, Islamic Azad University, Abdanan, Iran; 3 English Department, Shiraz University of Medical Sciences, Shiraz, Iran

**Keywords:** Academic self-efficacy, Learning-related emotions, Metacognitive strategies, Academic performance, Medical students

## Abstract

**Abstract:**

Recognition of the factors affecting the medical students’ academic success is one of the most important challenges and concerns in medical schools. Hence, this study aimed to investigate the mediating effects of metacognitive learning strategies and learning-related emotions in the relationship between academic self-efficacy with academic performance in medical students.

**Methods:**

The present study was carried out on 279 students of medicine studying at Shiraz University of Medical Sciences. The students filled out three questionnaires: academic emotions (AEQ), metacognitive learning strategies, and academic self-efficacy questionnaires. The data were analyzed using SPSS and Smart PLS3.

**Results:**

The results of structural equation modeling revealed that the students’ self-efficacy has an impact on their learning-related emotions and metacognitive learning strategies, and these, in turn, affect the students’ academic performance. Moreover, learning-related emotions influence the metacognitive learning strategies, which in turn mediate the effect of emotions on academic performance.

**Discussion:**

The results of this study revealed that metacognitive strategies and learning-related emotions could play a mediating role in the relationship between students’ self-efficacy and academic performance.

## Background

Academic success and obtaining good grades are among the main goals in all levels of education while having positive outcomes both for the learners and educational systems. Therefore, identifying the factors influencing the students’ academic success has ever been one the most important concerns of the researchers and educational psychologists [1], and also one of the challenges faced by medical schools [2, 3]. To this end, researchers have focused on recognition of the role of motivation, learning strategies, and academic emotions in the students’ learning and performance [4–7].

However, most of the researches have been conducted using correlation analysis [6], qualitative methods [4], and experimental approaches [8]; they have revealed a positive and simple relationship between these variables and academic performance [9] and have not shown direct and indirect effect of these variables on each other. Moreover, most of these studies have been carried out in the field of psychology, social sciences, and education [10, 11], and the results of these studies cannot be generalized to the medical context. Since the nature of the academic field is supposed to affect the students’ learning strategies [12], there may be a difference between medical students’ learning approaches in comparison with those of other students in higher education [13]. Moreover, students in different academic settings and environments have revealed to experience different emotions. By implication, emotions might be different across these contexts [5]. As Artino et al. noted, these emotional factors had almost been neglected in medical education literature. Instead, medical education literature tends to focus mostly on cognitive factors such as prior academic achievement, which do not explain much of the variance in academic outcomes [2]. Yet, a large body of medical literature on emotions indicates that many medical students experience stressful situations during their education resulting in depression and anxiety. There has been very little attempt to look at how these emotions influence the students’ self-regulating learning (SRL) [2]. As to the Iranian context, since the physicians have a very high income, most students are eagerly competing to be admitted in this major, so the smartest students with the highest potential get accepted to continue their studies in this major.

On the one hand, there is still limited knowledge about the effect of motivation and emotions on the students’ academic outcomes both in the classroom and clinical settings [2, 14]. On the other hand, most of the studies have been conducted in western countries [15], and generalizing their findings to other countries, especially developing ones, has been criticized [16]. Therefore, this study was conducted to investigate the effect of self-efficacy, learning-related emotions, and metacognitive learning strategies on medical students’ academic performance. More specifically, it was attempted to determine how metacognitive learning strategies can mediate the relationship between self-efficacy and positive learning-related emotions and academic performance.

## The effect of positive academic emotions on academic performance

Emotion is a subjective status accompanied by physiological reactions and responses to some conditions, actions, and events. Pekrun (2006) defines academic emotions as those which are directly related to achievements, activities, and outcomes [17]. This term was first used by Pekrun et al. (2002) in the field of education [4] classified into positive (enjoyment, pride, hope); negative (boredom, anger, anxiety); activating (joy, pride, anger); and deactivating (shame) emotions [5, 17]. Emotions have complex associations with cognitive, motivational, and behavioral processes, especially in the classroom and educational settings [4, 5, 14, 17, 18], in all educational situations (before, during, and after attending the classroom, studying and testing) [4, 10], and in clinical settings [2, 14], as experienced by the students.

Moreover, some researchers have considered emotions as a significant factor directly or indirectly associated with learners’ achievements; satisfaction; physical and mental health; motivation; learning strategies; cognitive sources; self-directed learning; quality of teacher-learner interactions; class education; concentration; information processing, storing, retrieving, and learning; and consequently academic achievement [1, 2, 4, 5, 10, 17, 19, 20]. Pekrun (2006) indicated that pleasant positive emotions like enjoyment positively influences on academic achievement. On the contrary, unpleasant deactivating emotions like boredom can reduce our motivation and disturb data processing, showing the negative effect of such emotions on academic achievement [17].

Chin et al. (2017) found a significant relationship between the students’ positive emotions and their performance [21]. Pekrun et al. (2009) revealed that positive activating emotions like enjoyment, hope, and pride have a significant relationship with the students’ midterm exam scores [10]. Generally, previous research showed that positive emotions such as enjoyment, hope, and pride are predictors of academic achievement [4, 5].

## The effect of academic self-efficacy on academic performance

Academic self-efficacy is one of the important factors influencing academic performance. Academic self-efficacy refers to the students’ beliefs and attitudes toward their capabilities to achieve academic success, as well as belief in their ability to fulfill academic tasks and the successful learning of the materials [22, 23].

Self-efficacy beliefs lead to the individuals’ excellent performance through increasing commitment, endeavor, and perseverance [24]. The learners with high levels of self-efficacy attribute their failures to lower attempts rather than lower ability, while those with low self-efficacy attribute their failure to their low abilities [25]. Therefore, self-efficacy can influence the choice of tasks and perseverance while doing them. In other words, students with low self-efficacy are more likely to be afraid of doing their tasks, avoiding, postponing, and give them up soon [22, 23].

In contrast, those with high levels of self-efficacy are more likely to rely on themselves when faced with complex issues to find a solution to the problem, as well as being patient during the process, making more efforts, and persisting longer to overcome the challenges [9, 23, 26]. Therefore, it seems that self-efficacy is one of the most important factors in the students’ academic success. For example, Chemers and Garcia found that the students’ self-efficacy in the first year of university is a strong predictor of their future performance [27].

Alyami et al. (2017) conducted a study on 214 university students and revealed that academic self-efficacy has a positive and significant effect on their academic performance [28]. Other studies have shown that academic self-efficacy has a considerable effect on the students’ learning, motivation, and academic performance [9, 18, 29–31].

## The effect of metacognitive learning strategies on academic performance

In the recent years, self-regulated learning and especially metacognitive learning strategies [32] have received a great deal of attention, and many studies are being conducted in this field [33]. Predominantly, metacognitive strategies are among the key components of self-regulated learning, enabling learners to plan, monitor, and regulate their cognition [34, 35]. Today, it is believed that learners using more metacognitive learning strategies effectively have better study plans, more efficiently monitor and evaluate their learning and perception of the materials, are more responsible, find and solve their problems, and try hard to learn deeply [36, 37]. They certainly succeed more than their peers with no skills in the use of such strategies [38]. In this regard, it has been confirmed that metacognitive learning strategies have a main role in academic success, as shown by the theories and researches [1, 4, 23, 24, 35, 38].

## Conceptual framework and hypotheses

The control- value theory of achievement emotions is a comprehensive framework for analysis of the effects of emotions on the students’ academic performance. As hypothesized by Pekrun, in this theory, positive emotions influence the students’ achievement indirectly through the mediating role of cognitive, metacognitive, and self-regulating behaviors [17, 19].

Generally speaking, emotions can influence the students’ achievement through two main pathways of cognitive and motivational and four mechanisms. In the cognitive pathway, emotions can influence one’s performance through three mechanisms, including mood-dependent memory, and cognitive and metacognitive learning strategies, and the use of cognitive sources [14, 39].

In contrast, positive emotions resulting from the use of deep, flexible, and complex learning strategies and self-regulation facilitate the individuals’ learning [4], so that the students who experience positive emotions utilize deeper strategies and more metacognitive processing [4, 40], that, in turn, enhances the students’ achievement. Therefore, the effect of emotions on academic performance can be mediated by the use of metacognitive learning strategies.

Based on Pekrun’s control-value theory [17, 40], cognitive assessment is supposed to be one of the significant antecedents of academic emotions categorized into control assessments (perceived control) and value assessments (perceived value). Control assessments are related to the individuals’ perception of the controllability of achievement activities and their consequences. These assessments are shown through our expectations and perception of competence, such as self-efficacy. Therefore, academic self-efficacy (as a cognitive assessment) can influence academic emotions [1, 4, 14]. On the other hand, many researchers have investigated the role of self-efficacy in academic achievement since the introduction of the concept of self-efficacy by Bandura (1977) [9, 18, 30, 31]. Bandura’s (1977) social cognitive theory discusses self-efficacy as the main construct, which affects both performance and motivation [26].

Some researchers believe that a part of the relationship between self-efficacy and academic achievement can be attributed to metacognitive learning strategies [35, 41]. More specifically speaking, evidence shows that students with higher self-efficacy (as an expectancy component) show more endeavor and perseverance when faced with challenging situations [23]. Despite the positive effect of self-efficacy on the amount of attempt, evidence shows that the quality of the efforts of self-efficacious students is different as well; such students use various deeper cognitive and metacognitive processing strategies compared to their peers with lower self-efficacy. This leads to better learning and academic achievement [35, 38]. On the contrary, students with low self-efficacy seek easier tasks to avoid failure and use superficial strategies while disregarding deep learning [6].

Therefore, as shown in other studies, self-efficacy and metacognitive learning strategies are closely related [35, 42]. As stated by Pintrich, self-efficacy becomes a key determinant of whether learners adopt these strategies or not. According to self-regulated learning theories, apart from being aware of the cognitive and metacognitive strategies, students should be motivated to enthusiastically use these strategies to succeed [35]. In this respect, the general expectancy-value theory of motivation [35, 43] suggests that there are three motivational components that might be associated with the components of self-regulated learning like metacognitive strategies: (a) an affective component, which involves emotional reactions of students to the task (pride, anger, etc.), (b) an expectancy component, including the students’ beliefs about their capability to do a task (self-efficacy), and (c) a value component, including the students’ goals and beliefs about the importance and interest of the task. Prior research reveals that the expectancy, value, and affective components are positively associated with the self-regulated learning components [35, 44].

**Fig. 1:**
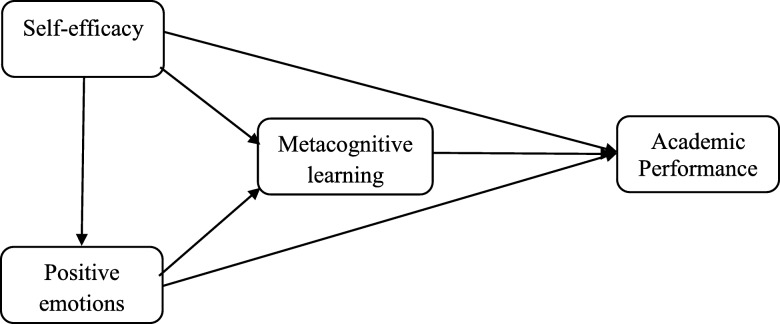
The conceptual model

In short, the studies conducted in this field have shown a positive association between self-efficacy and metacognitive learning strategies [35, 36, 42, 45]. On the other hand, many studies have indicated that metacognitive learning strategies are one of the most important predictors of the students’ academic success [4, 9, 24, 35, 38, 46]. Therefore, as shown in some studies, metacognitive learning strategies mediate the effect of self-efficacy on academic performance [47]. There has been some progress in research in this area. According to the review of the literature, although many studies have been conducted on direct effect of variables as academic emotions, academic self-efficacy, metacognitive learning strategies and their roles in academic achievement, few studies have focused on direct and indirect relationship among these variables and investigated the role of emotions, self-efficacy, and metacognitive learning strategies together as predictors of academic achievement in a structural equation model. Previous studies have either investigated the effect of the above-mentioned variables on each other separately [2, 36, 42, 48], or they have focused on fields other than medical education [1, 4, 10, 28, 32, 46]. Therefore, according to the control-value theory [40], the expectancy-value theory of motivation [43], the social cognitive theory and review of the literature, the present study was designed to test the following research hypotheses and conceptual model (see Fig. 1):

## Hypotheses

- H1: Academic self-efficacy has a direct effect on academic performance.
- H2: Positive academic emotions have a direct effect on academic performance.
- H3: Metacognitive learning strategies have a direct effect on academic performance.
- H4: Academic self-efficacy has a direct effect on positive academic emotions.
- H5: Academic self-efficacy has a direct effect on metacognitive learning strategies.
- H6: Positive academic emotions have a direct effect on metacognitive learning strategies.
- H7: Metacognitive learning strategies mediate the relationship between academic self-efficacy and academic performance.
- H8: Metacognitive learning strategies mediate the relationship between positive academic emotions and academic performance.
- H9: Positive academic emotions mediate the relationship between academic self-efficacy and academic performance.

## Methods

### Participants and procedures

This cross-sectional study was conducted on 279 (64.5% females and 35.5% males) medical students studying in their first to fifth semesters (basic sciences period) in the 2018–2019 academic years at Shiraz University of Medical Sciences, Iran. The response rate of the participants was 279/350 (79%).

In general, the course of medicine lasts for 7 years in Shiraz University of Medical Sciences, divided into four periods, including basic sciences, physiopathology, externship, and internship. Each year about 200 medical students enter Shiraz Medical School straight after graduation from high school. Students’ major courses were investigated in each semester. In this study, the pathology, anatomy, cardiovascular system, digestive system, urinary tract, glands, reproductive system, blood, musculoskeletal system, neurology, respiration, and head and neck anatomy courses were selected. The mentioned courses are presented both theoretically or integration of theory and practice.

The students aged between 18 and 35 years old (mean 19.6, SD 3.2). Although a sample size of over 200 is satisfactory for conduction of Structural Equation Modeling (SEM), some researchers have suggested 20 subjects for each variable [49, 50]. Our sample size satisfied both views. Of course, the advantage of Partial Least Square (PLS) approach is that it requires a smaller sample size in comparison with other approaches such as LISREL and AMOS. PLS is more suitable for real applications, especially in the case of more complex models, the use of this approach is more satisfactory.

The subjects were selected using the convenience sampling method. Since this study was conducted on humans, first, it was approved by the Research Ethics Committee of Shiraz University of Medical Sciences with the code of IR.SUMS.REC.1397.595. Also, the students were assured that their information would remain confidential. Before commencement of the study, written informed consent was obtained from all the students.

They were asked to fill out the forms anonymously. Participation in the study was completely voluntary, and those who were willing to participate filled out the questionnaires. The questionnaires were distributed among the students, and they were asked to answer the questions about how they experienced these emotions during the semester. Also, the questionnaires of metacognitive learning strategies and academic self-efficacy were simultaneously distributed.

## Measures

In the current study, three types of questionnaires were applied.

## Academic emotions questionnaire (AEQ)

AEQ developed by Pekrun et al. is a valid and reliable questionnaire measuring the students’ academic emotions [5]. It consists of 3 parts measuring the emotions related to the classroom, learning, and exams separately. In this study, an adapted version of AEQ was used to evaluate the students’ experienced positive emotions while studying (positive learning-related emotions).

This subscale includes three positive emotions related to learning (enjoyment, pride, hope) with 22 questions answered in the form of a 5-point Likert scale ranging from completely disagree [1] to completely agree [5]. Pekrun et al. have reported a good validity and reliability coefficient for this questionnaire [5].

## Metacognitive learning strategies questionnaire

Motivated Learning Strategies Questionnaire (MLSQ) is a valid and reliable questionnaire used for evaluation of the students’ motivational orientations and self-regulatory learning strategies [44]. This questionnaire contains two subscales of motivation and self-regulated learning and has been used in many studies in medical and other fields [2]. In this study, metacognitive learning strategies subscale consisting of 12 items was used, in which results are scored using 5 -point Likert scale. Pintrich et al. have reported a Cronbach’s alpha of 0.79 for this subscale [44].

## Academic self-efficacy questionnaire

The self-efficacy for learning and performance is one of the subscales of the above-mentioned questionnaire (MLSQ). It contains 8 questions evaluating the students’ beliefs regarding their abilities and performance.

These items are scored using a 5 -point Likert scale. Pintrich et al. also reported a desirable validity and reliability for this instrument [44], and it has been used in many studies [6, 9].

## Academic performance

To assess the students’ academic performance, their final exam scores in that semester were considered. Scores in a course which are obtained on the midterm and final exams and also semester-work component consisting of a term paper, quizzes, and assignments were all considered as indicators of academic performance. The assignments include class presentation individually or in group, review of a book or paper, group discussions which are done as a part of the course requirements. Also, some of the lecturers assign some research projects which are conducted are conducted and presented by students in the class. In addition, in the context of Shiraz University of Medical Sciences, the students are assessed through formative and summative multiple-choice tests.

We used SPSS version 21 to calculate the mean and standard deviation and correlation coefficients between the variables. Also, we used Smart-PLS 3 to determine the validity and reliability and also the path coefficients between the variables. There are two types of structural equation modeling (SEM) namely covariance-based SEM (CB-SEM) and partial least squares SEM (PLS-SEM). For the current study, PLS-SEM applying smart-PLS software was selected which empowers the researchers to estimate very complex models with many constructs and indicator variables, especially when prediction remains the main goal of the analysis. PLS-SEM basically offers more flexibility regarding data requirements and specification of the associations between the constructs and indicator variables [51]. PLS-SEM focuses on two processes, including the measurement model and structural model [52].

## Results

The matrix correlation results showed that, self-efficacy has a significant and positive relationship with academic performance (r = 0.46, *p* ≤ 0.01), metacognitive learning strategies (r = 0.59, *p* ≤ 0.01), and positive learning-related emotions (r = 0.65, *p* ≤ 0.01). In addition, findings showed that, positive learning-related emotions have a positive and significant correlation with metacognitive learning strategies (r = 0.55, *p* ≤ 0.01) and academic performance (r = 0.48, *p* ≤ 0.01). Also, as shown in Table 1, a significant and positive correlation was found between metacognitive learning strategies and academic performance (r = 0.45, *p* ≤ 0.01).

**Table 1:** Relationship between academic performance, self-efficacy, metacognitive learning strategies, and positive emotions

	Mean	SD	1	2	3	4
1- Academic performance	16.95	1.433	1			
2- Self-efficacy	2.68	1.041	0.46^**^	1		
3- Metacognitive learning strategies	2.89	0.990	0.45^**^	0.59^**^	1	
4- Positive learning -related emotions	2.97	0.945	0.48^**^	0.65^**^	0.55^**^	1

*p < .05 **p < .01

## The measurement model

The measurement model in PLS was evaluated in terms of internal consistency reliability, convergent validity, and discriminant validity. Internal consistency reliability measures the degree to which the items measure latent construct (Hair et al., 2006), assessed through composite reliability scores. Composite reliability of 0.7 or greater is considered acceptable. Results showed that the CR scores of all constructs exceeded the recommended criterion of 0.7, demonstrating appropriateness of the scales used in the current study.

Next, the factor loadings and Average Variance Extracted (AVE) were assessed to determine the convergent validity of the constructs. Individual item loadings greater than 0.7 are considered as adequate. Based on the results of the measurement model (Table 2), all the construct items exhibited loadings exceeding 0.7 with adequate AVE ranging from 0.68 to 0.79. Results also showed adequate discriminant validity as all the square roots of AVE were higher than the inter-correlation value between the constructs (Table 3). Therefore, the reliability and validity of the research constructs were confirmed.

**Table 2:** The results of confirmatory factor Analysis: Factor loadings, composite reliability (CR), and average variance extracted (AVE)

Construct	items	loadings	CR	AVE	Convergent Validity
Enjoyment	q1	0.94	0.97	0.82	Yes
	q2	0.93			
	q3	0.91			
	q4	0.89			
	q5	0.95			
	q6	0.94			
	q7	0.87			
	q8	0.83			
	q9	0.82			
	q10	0.97			
Hope	q11	0.94	0.98	0.89	Yes
	q12	0.93			
	q13	0.96			
	q14	0.92			
	q15	0.97			
	q16	0.93			
Pride	q17	0.95	0.97	0.88	Yes
	q18	0.94			
	q19	0.92			
	q20	0.93			
	q21	0.96			
	q22	0.93			
Self-efficacy	q23	0.94	0.98	0.88	Yes
	q24	0.90			
	q25	0.95			
	q26	0.93			
	q27	0.96			
	q28	0.91			
	q29	0.97			
	q30	0.94			
Metacognitive learning strategies	q31	0.90	0.98	0.87	Yes
	q32	0.95			
	q33	0.98			
	q34	0.70			
	q35	0.96			
	q36	0.95			
	q37	0.93			
	q38	0.98			
	q39	0.92			
	q40	0.95			
	q41	0.98			
	q42	0.97

**Table 3:** Discriminant validity coefficients of the research constructs

construct	Positive emotions	Self-efficacy	Metacognition
Positive learning-related emotions	**0.97**		
Self-efficacy	0.55	**0.94**	
Metacognitive learning strategies	0.65	0.59	**0.93**

## The structural model

**Fig. 2:**
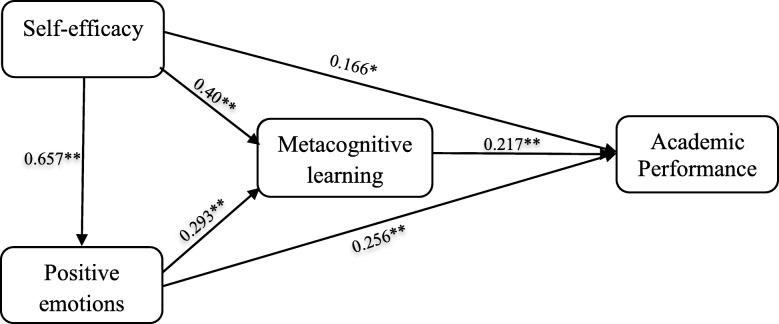
SEM depicting relationships between metacognitive learning strategies, positive learning-related emotions and academic self-efficacy with academic performance. ** indicates statistically significant at *p* < 0. 01 level and * shows statistically significant at *p* < 0. 05 level. Values for each arrow indicate the standardized path coefficients

Structural model assessment was used to test hypothesized theoretical relationships in the suggested conceptual framework, which included the relationship between positive emotions, self-efficacy, metacognitive learning strategies, and academic performance (Figs. 1, 2). The coefficient of determination (R^2^ values) and path coefficients (beta values) were the parameters used to determine how well the data supported hypothesized relationships. Also, PLS path-analysis of bootstrapping was applied to find the path correlation between the research variables to understand whether the path coefficient is significant for hypothesized relationships.

**Fig. 3:**
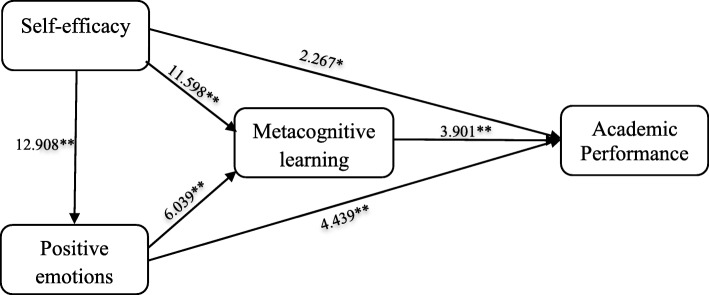
Path analysis of bootstrapping shows T-test scores related to path coefficients depicted in Fig. 2. T-test scores that are higher than 1.96 are significant at 0.05 level, and T-test scores that are higher than 2.58 are significant at 0.01 level

Figure 2 shows the path coefficients estimated from the PLS analysis. According to the results, hypotheses H1, H2, H3, H4, H5, H6, H7, H8, and H9 were all supported (Table 4). To determine significance of all the relationships in the model, bootstrapping procedure as a re-sampling technique was applied. Based on the estimated path coefficients showed in Fig. 2 and the t-test statistics scores indicated in Fig. 3, self-efficacy demonstrated a direct, positive, and statistically significant effect on academic performance (H1 *p* < 0.05), positive learning-related emotions (H4 *p* < 0.001), and metacognitive learning strategies (H5 *p* < 0.001). Similarly, positive emotions had a direct, positive, and statistically significant effect on academic performance (H2 *p* < 0.001) and metacognitive learning strategies (H6 *p* < 0.001). Also, as hypothesized, metacognitive learning strategies had a direct, positive, and statistically significant effect on academic performance (H3 *p* < 0.001) (Figs. 2 and 3).

**Table 4:** Path coefficients for hypothesis testing

H	Path	*B*eta	t	*P*. value	Decision
H_1_	self-efficacy→academic performance	0.166	2.29	0.014	Supported
H_2_	positive emotions→academic performance	0.256	4.44	< 0.001	Supported
H_3_	metacognition→academic performance	0.217	3.90	< 0.001	Supported
H_4_	self-efficacy→positive emotions→	0.657	12.91	< 0.001	Supported
H_5_	self-efficacy→metacognition	0.400	11.59	< 0.001	Supported
H_6_	positive emotions→metacognition	0.293	6.04	0.001	Supported
H_7_	self-efficacy→metacognition→academic performance	0.09	3.21	< 0.001	Supported
H_8_	positive emotions→metacognition→academic performance	0.063	3.62	< 0.001	Supported
H_9_	self-efficacy→positive emotions→academic performance	0.168	3.75	< 0.001	Supported

Moreover, the mediation test indicated an indirect effect of self-efficacy on academic performance through metacognitive learning strategies (H7) (b = 0.09, t = 3.77, *p* < 0.001), and positive learning-related emotions (H9) (b = 0.168, t = 3.50, *p* < 0.001). Similarly, the mediation test indicated an indirect effect of positive learning-related emotions on academic performance through metacognitive learning strategies (H8) (b = 0.063, t = 3.27, *p* < 0.001).

The goodness-of-fit R^2^ of latent endogenous variables can be applied to assess the utility of the proposed model. In the proposed model, 30% of the variance in academic performance was explained by self-efficacy, positive emotions, and meta-cognitive learning strategies. Moreover, results indicated that 40% of the variance in metacognitive learning strategies was explained by self-efficacy and positive emotions. Furthermore, findings showed that 43.2% of the variance in positive emotions was explained by self-efficacy.

Hair et al. (2014) also suggested reporting on predictive relevance (Q^2^) besides basic parameters. According to Fornell and Cha (1994), the model has predictive quality if the cross-redundancy value is found to be more than 0; otherwise, predictive relevance (Q^2^) of the model cannot be achieved. Based on the results obtained using Smart PLS 3.0 software, obtained cross-validated redundancy was found to be 0.289, 0.422, and 0.389 for academic performance, positive emotions, and meta-cognitive learning strategies, respectively.

## Discussion

According to Pekrun et al. (2007), in control-value theory, it is supposed that the students’ cognitive appraisal, like self-efficacy affects positive emotions as a personal factor, which also impacts the students’ academic achievement through a cognitive path (metacognitive strategies).

Our results strongly supported predictive links among academic self-efficacy, positive emotions, metacognitive learning strategies, and academic performance.

First, the findings of the study demonstrated the influence of academic self-efficacy on positive emotions. As mentioned above, based on Pekrun’s control-value theory [17, 40], cognitive assessment is supposed to be one of the significant antecedents of academic emotions categorized into control assessments (perceived control) and value assessments (perceived value). Control assessments are related to the individuals’ perception of the controllability of achievement activities and their consequences. These assessments are shown through our expectations and perception of competence, such as self-efficacy. Therefore, academic self-efficacy (as a cognitive assessment) can influence academic emotions [14]. It can be expected that, when the students believe in their ability to perform their tasks successfully, they will enjoy the learning process more; also, it seems reasonable that these individuals experience more feelings like hope and pride compared to the students with low self-efficacy. The findings of some studies indicated a positive relationship between academic self-efficacy and positive emotions [1, 5].

The second finding of this study was the influence of academic self-efficacy on metacognitive learning strategies. One of the key determinants of the use of metacognitive learning strategies by the learners is their self-efficacy. Despite the positive effect of self-efficacy on the amount of attempt, evidence shows that the quality of the efforts of self-efficacious students is different as well; such students use deeper various metacognitive processing strategies compared to their peers with lower self-efficacy [6]. Previous studies have revealed that self-efficacious students use more metacognitive learning strategies compared to their peers [9, 35–37, 42, 45]. Pintrich and De Groot believe that the students who trust in their abilities are more likely to be self-efficacious and try to recognize their academic tasks and plan for their educational affairs. Quality of the efforts and the use of a variety of deep cognitive and metacognitive learning strategies are different in such students compared to their peers [35].

The findings of other studies revealed a significant relationship between positive academic emotions and metacognitive learning strategies. Specifically, control-value theory predicts that achievement emotions influence the use of metacognitive learning strategies [17, 40]. Pekrun et al. believed that positive emotions resulted from the use of deep, flexible, and complex learning strategies and that self-regulation facilitated the individuals’ learning [4], so that the students who experience positive emotions utilized deeper strategies and more metacognitive processing [4, 40]. This, in turn, enhances the students’ achievement. The results of some studies have confirmed a positive association between positive academic emotions and cognitive and metacognitive learning strategies [1, 4, 6, 48]. As assumed in our model, positive academic emotions positively predicted academic performance. This is in line with the results of previous studies [1, 2, 4, 6, 10, 21, 30, 46]. Pekrun et al. stated that emotions are involved in almost all aspects of the teaching and learning process [40]. Positive emotions related to learning can influence the learners’ performance through effects on the quality of the learning process, quality of teacher-learner and peer-peer relationships in the classrooms, and effective teaching [19]. In this regard, Meinhardt and Pekrun stated that learning is continuously accompanied by emotions and emotions influence concentrating, processing, storing, and retrieving of the information [20]. In summary, emotions deal with four types of essential mental processes including attention, concept formation, and allocation of cognitive and metacognitive sources which are necessary for learning. Evidence shows the effect of emotions on cognitive performance. Also, based on Pekrun’s control-value theory, emotions can influence the individuals’ academic performance through their effect on some mediating factors such as academic motivation, memory, and cognitive and metacognitive sources [40].

Another finding of this study was a significant relationship between metacognitive learning strategies and academic performance. Scholars believed that the students who use more effective metacognitive learning strategies have better study plans, monitor, and evaluate their learning and perception of the materials more efficiently, assume their responsibility, detect and solve their problems, and try hard to learn deeply [36, 53]. They surely achieve more than their peers who are not skillful in the use of such strategies [38]. In this respect, the role of metacognitive learning strategies has been well confirmed in academic success by the theories and researches [1, 4, 23, 24, 35, 38, 41, 46]. Finally, as assumed in our model, findings showed that the influence of self-efficacy on academic performance depends on multiple relationships and interplay of positive emotion and metacognitive learning strategies. In particular, self-efficacy positively influences academic performance when it is mediated by positive emotion and metacognitive learning strategies. Based on the control-value theory, self-efficacy can act as an antecedent of emotion, meaning that academic emotion can mediate the effect of self-efficacy on academic performance [14, 17]. Thus, it seems reasonable to assume that the students who believe in their own capabilities to learn and perform some of their scientific tasks enjoy learning new materials more than the others. Since these students believe that they have the necessary abilities to learn their materials, they have a sense of pride while learning. Also, since they believe in their abilities, they are optimistic about their learning and also the materials to be learned. Therefore, it is concluded that highly self-efficacious students experience more positive emotions while studying and learning, which can, in turn, lead to better academic performance.

Results also showed a significant indirect effect of positive emotions on the students’ academic performance, so that metacognitive learning strategies mediated the relationship between the students’ positive academic emotions and their academic performance. This finding is consistent with the results of other studies [1, 4]. Also, based on Pekrun’s control-value theory, emotions can influence the individuals’ academic performance through their effect on some mediating factors such as metacognitive sources [14, 40]. For instance, King and Areepattamannil (2014) found a significant and positive relationship between positive emotions and metacognitive learning strategies (planning, monitoring, regulating) [48]. Somehow the students who experience positive emotions in the learning process are more inclined to use flexible, complex, and self-regulatory learning strategies; in general, emotions bring about more involvement, and the use of deeper processing strategies consequently leads to better performance [4]. Therefore, positive emotions are not enough to guarantee academic achievement by themselves since metacognitive learning strategies are also necessary.

## Conclusion

In conclusion, our theoretical model implies the antecedents and consequences of positive academic emotion, especially metacognitive learning strategies and academic performance. Our results revealed that the students who believed in their abilities and had more positive emotions used more metacognitive learning strategies, resulting in better academic performance.

## Limitations

In general, this study can be regarded as evidence regarding the direct and indirect effects of self-efficacy and positive academic emotions on the medical students’ academic performance; it also supports the control -value theory and other studies conducted in this field. Despite these strengths, this study had some limitations. First, this study was a cross-sectional quantitative study, so it was not possible to precisely show the cause and effect relationship between the variables. Second, in this study, self-report questionnaires were used that raises the possibility of response bias. However, the use of self-report questionnaires enables us to elicit the participants’ beliefs and personal perceptions toward their learning process. Lastly, convenience sampling method was used, which does not reveal random sampling features and makes generalization of the results impossible.

## Implications

Teachers in medical schools can reduce the students’ stress through providing supportive and calm environments since competitive and stressing contexts influence the students’ self-efficacy; they can invigorate positive emotions in the students by giving appropriate, positive, and supportive feedbacks, creating interactive approaches in the classrooms, and encouraging the students to cooperate in class discussions instead of competition. Since the teacher’s enthusiasm, positive feedback to success, cooperation, sense of belonging to class are positively related to the students’ enjoyment of learning and hope for success in learning.

Results of the study also suggest that teachers in medical schools should take measures in order to create a peaceful environment where the students feel comfortable and secure since positive feeling toward the learning climate and environment can increase positive emotions like enjoyment, pride, and hope in the students while learning, leading to academic success.

In addition, creating a climate in which the students experience freedom and respect would make them enjoy their presence in the class and learning which in turn leads to involvement in teaching, more academic engagement, and the use of deeper learning strategies.

Moreover, some factors can influence academic emotions indirectly. For example, quality of teaching in the classroom can directly influence the students’ dominance, perceived academic control, and self-efficacy, which in turn influences their emotions indirectly. Thus, behavior in the class, expressed emotions, and the teachers’ quality of teaching can influence the students’ learning which, in turn, can be a significant factor in raising the students’ positive emotions and self-efficacy.

## Data Availability

The datasets used during this study are available from the corresponding author on reasonable request.
